# Subcutaneous administration of casein attenuates atherosclerotic progression in male apoE^‐/‐^ mice fed with high‐fat diet

**DOI:** 10.1002/fsn3.1638

**Published:** 2020-06-07

**Authors:** Lingbo Hou, Yuting Wang, Mingjing Zhang, Yijun Yu, Ye Gu

**Affiliations:** ^1^ Department of Cardiology Wuhan Fourth Hospital Puai Hospital Tongji Medical College Huazhong University of Science and Technology Wuhan China

**Keywords:** atherosclerosis, Casein, fibrosis, inflammation

## Abstract

The impact of casein on atherosclerotic lesion progression remains controversial. In this study, we tested the effect of casein on atherosclerotic development and its potential mechanisms in male apolipoprotein E knockout (apoE^‐/‐^) mice fed with high‐fat diet (HFD). Male apoE^‐/‐^ mice fed with HFD were randomized into HFD group (subcutaneous injection with 0.5 ml of 0.9% sodium chloride daily, *n* = 6) and HFD + Casein group (subcutaneous injection with 0.5 ml of 10% casein daily, *n* = 6). Body weight was recorded at baseline and once a week thereafter. After 12 weeks of treatment, plasma lipid and inflammatory markers, and histological characterization of atherosclerotic plaques in the aortic arch and aortic sinus were analyzed. There was no significant difference in weight gain between the two groups after 12 weeks of treatment. Plasma levels of total cholesterol (TC), triglyceride (TG), and low‐density lipoprotein cholesterol (LDL‐C) were significantly lower, while high‐density lipoprotein cholesterol (HDL‐C) level tended to be higher in the HFD + Casein group compared with the HFD group. The positive percentages of atherosclerotic lesions in aortic arch and aortic sinus as well as collagen deposition in aortic sinus plaques were significantly lower in the HFD + Casein group compared with the HFD group. Plasma levels of interleukin (IL)‐1β and granulocyte‐macrophage colony‐stimulating factor (GM‐CSF) were also significantly lower in the HFD + Casein group compared with the HFD group. In conclusion**,** subcutaneous administration of casein attenuates atherosclerotic lesion progression, possibly through decreasing fibrosis and inflammatory responses in male apoE^‐/‐^ mice fed with HFD.

## Graphical abstract

The impact of casein on atherosclerotic lesion progression remains controversial. Our study finds that subcutaneous administration of 10% casein decreases plasma lipid concentrations in male apoE‐/‐ mice fed with high‐fat diet (HFD). Casein treatment is associated with attenuated atherosclerotic lesion progression, possibly by decreasing fibrosis and inflammatory responses in male apoE‐/‐ mice fed with HFD.

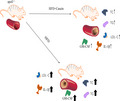

## 1 INTRODUCTION

Atherosclerosis progression could be modified by various dietary proteins, either positively or negatively (Torres, Guevara‐Cruz, Velazquez‐Villegas, & Tovar, 2015). As an important source of protein in the diet, the physiological role of milk protein has attracted increasing research interest beyond its nutritional value. It was reported that angiotensin‐I‐converting enzyme (ACE) inhibitory peptides existed in the amino acid sequences of milk protein (Saito, 2008), which could improve vascular elasticity and reduce blood pressure in spontaneously hypertensive rats (SHR) or hypertensive adults (Cadee et al., 2007; Sanchez et al., 2011; Xu, Qin, Wang, Li, & Chang, 2008). It was also found that milk protein intake could improve hyperglycemia by stimulating insulin secretion in individuals with metabolic syndrome or type 2 diabetes mellitus (T2DM) (McGregor & Poppitt, 2013; Pasin & Comerford, 2015). Moreover, milk protein was shown to exert antiobesity effects in diet‐induced obese male C57BL/6J mice (Lillefosse et al., 2013). In addition, a meta‐analysis of prospective cohort studies suggested that consumption of milk or milk products was negatively correlated with incidence of stroke (Elwood, Pickering, Givens, & Gallacher, 2010).

Casein comprises approximately 80% of the total milk protein (Dalziel, Young, McKenzie, Haggarty, & Roy, 2017), which is not only naturally present in dairy, but also popularly utilized as a protein supplement in its purified form of various nutrition products. Casein is often applied as an animal source of protein in experimental research as well. Till now, the role of casein in human health, especially on the lipid metabolism remains an issue of debate. Huff, Roberts, and Carroll (1982) found that casein enhanced triglyceride (TG) levels in plasma and atherosclerotic lesions development in the aortic arch region in male New Zealand white rabbits. Similarly, Damasceno et al. (2001) reported that casein feeding contributed to increasing plasma cholesterol and TG concentrations and the area of aorta atherosclerotic lesions in adult male New Zealand rabbits, while Ijaz et al. (2018) showed that casein diet for 12 weeks did not affect the lipid profile in male C57BL/6J mice when consumed with either low‐fat diet (LFD) or high‐fat diet (HFD). Another study conducted by Meinertz, Nilausen, and Faergeman (1990) suggested that the low‐cholesterol or cholesterol‐enriched casein diets could improve plasma lipid concentrations in healthy participants.

It is well known that inflammation is a key factor in the progression of atherosclerosis (Hedin & Matic, 2018). It was demonstrated that chronic systemic inflammation, characterized by the increase of serum levels of C‐reactive protein (CRP) and proinflammatory cytokines, such as tumor necrosis factor α (TNF‐α) and interleukin (IL)‐6, was closely related to pathogenesis of multiple metabolic disorders including insulin resistance, T2DM, obesity, and dyslipidemia in both humans and animal models (Burhans, Hagman, Kuzma, Schmidt, & Kratz, 2018; Cox, West, & Cripps, 2015; Lira, Rosa Neto, Antunes, & Fernandes, 2014; Lontchi‐Yimagou, Sobngwi, Matsha, & Kengne, 2013). Studies over the last several decades suggested that milk proteins had immunomodulatory effects but the results were mixed. It was reported that κ‐casein suppressed lymphocyte proliferation induced by T‐ and B‐cell mitogens (Cross & Gill, 2000). Panagiotakos, Pitsavos, Zampelas, Chrysohoou, and Stefanadis (2010) found a decreased level of inflammatory markers associated with the consumption of dairy products among healthy adults. However, Pal and Ellis (2010) demonstrated that casein supplementation had no significant influence on plasma inflammatory markers in overweight and obese individuals. This finding is in accordance with the study conducted by Bohl, Bjornshave, Gregersen, and Hermansen (2016) which showed that casein protein did not alter circulating inflammatory markers in abdominally obese adults. Till now, the effect of casein on atherosclerotic progression and its potential mechanism are not fully understood. In this study, we tested the impact of casein on the atherosclerotic development in apolipoprotein E knockout (apoE^‐/‐^) mice fed with HFD (1.25% cholesterol).

## 2 MATERIALS AND METHODS

### 2.1 Animals and study groups

Six‐week‐old male apoE^‐/‐^ mice on C57BL/6 background were purchased from The Animal Center of Beijing University and kept under standard laboratory conditions (12 hr light cycle, temperature 25°C), with free access to standard chow and drinking water. After 2 weeks of acclimatization, the mice were weighed and randomly divided into HFD group (*n* = 6, subcutaneous injection with 0.5 ml of 0.9% sodium chloride daily) and HFD + Casein group (*n* = 6, subcutaneous injection with 0.5 ml of 10% casein daily) and fed with HFD for 12 weeks. Male mice were chosen in this study to avoid the potential impact of periodic sex hormone fluctuation on atherosclerosis. Subcutaneous injection was used to evaluate the effect of casein in a fixed dose range. This study was approved by the Animal Care Committees of Wuhan Fourth Hospital. Experimental protocol was based on the ARRIVE guidelines and guidelines from the National Institutes of Health for animal care and use (NIH Publications No. 85–23, revised 1996).

### 2.2 Harvesting of tissue

Body weight was recorded at baseline and once a week thereafter for 12 weeks. After 12 weeks of treatment, the mice were anesthetized with pentobarbital sodium (40 mg/kg) after 6 hr of fasting. Blood samples were collected from retro‐orbital plexus into heparin‐coated tubes and then centrifuged to separate plasma. Both the heart and whole aorta were rapidly removed. All samples and tissues were snap‐frozen and stored at −80°C until analysis.

### 2.3 Measurements of lipid and inflammatory markers

The plasma lipid profiles of total cholesterol (TC), TG, low‐density lipoprotein cholesterol (LDL‐C), and high‐density lipoprotein cholesterol (HDL‐C) were measured using commercially available kits (Nanjing Jiancheng Bioengineering Institute, A111‐1, A110‐1, A113‐1, A112‐1, Nanjing, China). The absorbance was recorded at 510 nm for TC and TG, and 550 nm for LDL‐C and HDL‐C.

Plasma levels of IL‐1β, IL‐6, and granulocyte‐macrophage colony‐stimulating factor (GM‐CSF) were analyzed using the ELISA kits (Neobioscience, EMC111, Shenzhen, China) according to the manufacturer's protocols. Plasma CRP level was measured with the ELISA kit (Elabscience, Wuhan, China) following the manufacturer's instructions. The absorbance was recorded at 450 nm.

### 2.4 Histological characterization of aortic arch and aortic sinus

Method used to evaluate the atherosclerosis in this model was identical as the method we used previously (Gao et al., 2015)**.** The aortic arch was dissected and opened longitudinally. The atherosclerotic lesions of aortic arch were quantified by en face Oil Red O staining and the percentage of Oil Red O positive area was measured using Image‐Pro Plus software (IPP, Media Cybernetics, Inc., Rockville, MD, USA).

Each heart was cut along with a horizontal plane between the lowest tips of the right and left atria. The upper portion was snap‐frozen in O.C.T. compound (Tissue‐Teck, 4,583, Torrance, U.S.A). Serial cryosections (7 μm thick) of the aortic sinus, spanning the three cusps of the aortic valves, were cut. Atherosclerotic lesions and collagen contents in plaques were determined by Oil Red O staining and Masson's Trichrome staining, respectively. Images were captured by microscope (Leica, DMi8, Germany) with 40 magnifications, and the stained area was measured using IPP software.

### 2.5 Statistical analysis

Data were expressed as mean ± standard deviation (*SD*). Normal distribution was assessed by the Kolmogorov–Smirnov test. Unpaired Student's *t* test was used to evaluate statistical differences between groups. A value of *p* < .05 was considered statistically significant. All analyses were performed using SPSS 21.0 software (SPSS Inc., Illinois, USA).

## 3 RESULTS

### 3.1 Body weight and plasma lipid parameters of HFD group and HFD + Casein group

**FIGURE 1:**
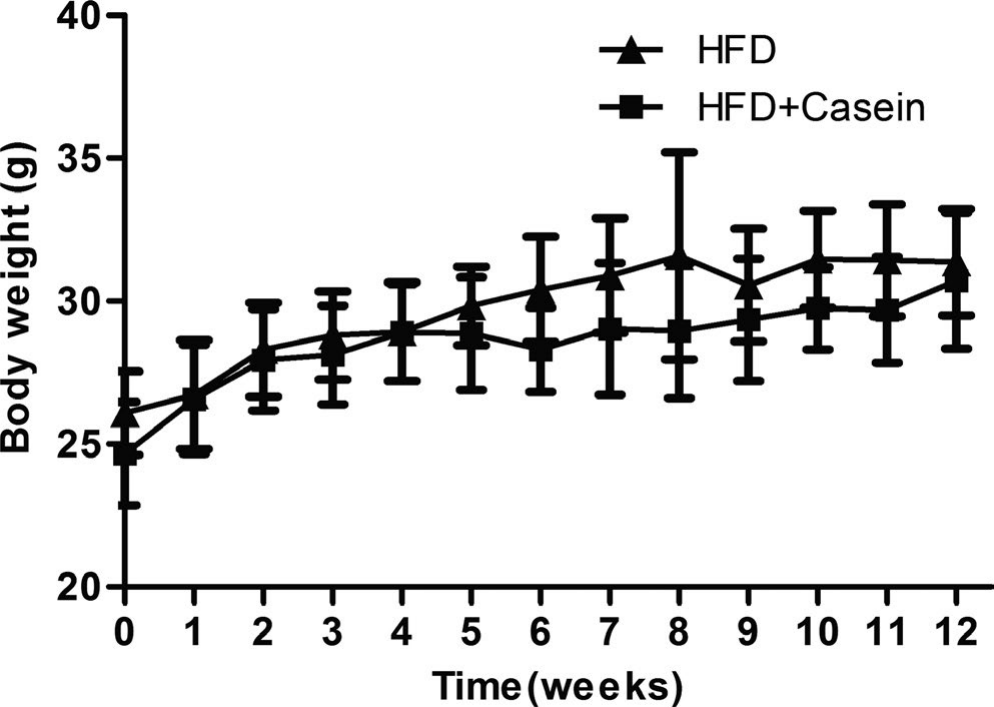
Body weight at each week for 12 weeks. ApoE^‐/‐^ mice were fed with high‐fat diet for 12 weeks without (HFD group) or with 10% casein injections (HFD + Casein group). The body weight was measured once a week for 12 weeks

Baseline and final body weight as well as body weight gain were similar between the HFD group and HFD + Casein group (Table 1, Figure 1). Plasma levels of TC, TG, and LDL‐C were significantly lower, while HDL‐C level tended to be higher in the HFD + Casein group compared with the HFD group (Table 1).

**Table 1:** Body weight and plasma lipid parameters of HFD group and HFD + Casein group

	HFD group (*n* = 6)	HFD + Casein group (*n* = 6)
Baseline body weight (g)	26.08 ± 1.46	24.67 ± 1.82
Final body weight (g)	31.37 ± 1.86	30.62 ± 2.13
Body weight gain (g)	5.28 ± 1.62	5.95 ± 3.07
TC (mmol/L)	39.93 ± 7.98	17.04 ± 4.82**
TG (mmol/L)	3.79 ± 1.09	2.07 ± 0.79*
LDL‐C (mmol/L)	8.88 ± 1.60	3.49 ± 1.98**
HDL‐C (mmol/L)	1.59 ± 0.75	2.58 ± 0.80

Note

ApoE^‐/‐^ mice were fed with high‐fat diet without (HFD group) or with 10% casein injections (HFD + Casein group). The body weight was measured once a week for 12 weeks. After 12 weeks of treatment, blood samples were collected and the plasma lipid profiles were measured. HFD, high‐fat diet; TC, total cholesterol; TG, triglyceride; LDL‐C, low‐density lipoprotein cholesterol; HDL‐C, high‐density lipoprotein cholesterol.

* *p* < .05;

** *p* < .01.

### 3.2 Histological characterization of aortic arch and aortic sinus of HFD group and HFD + Casein group

**FIGURE 2:**
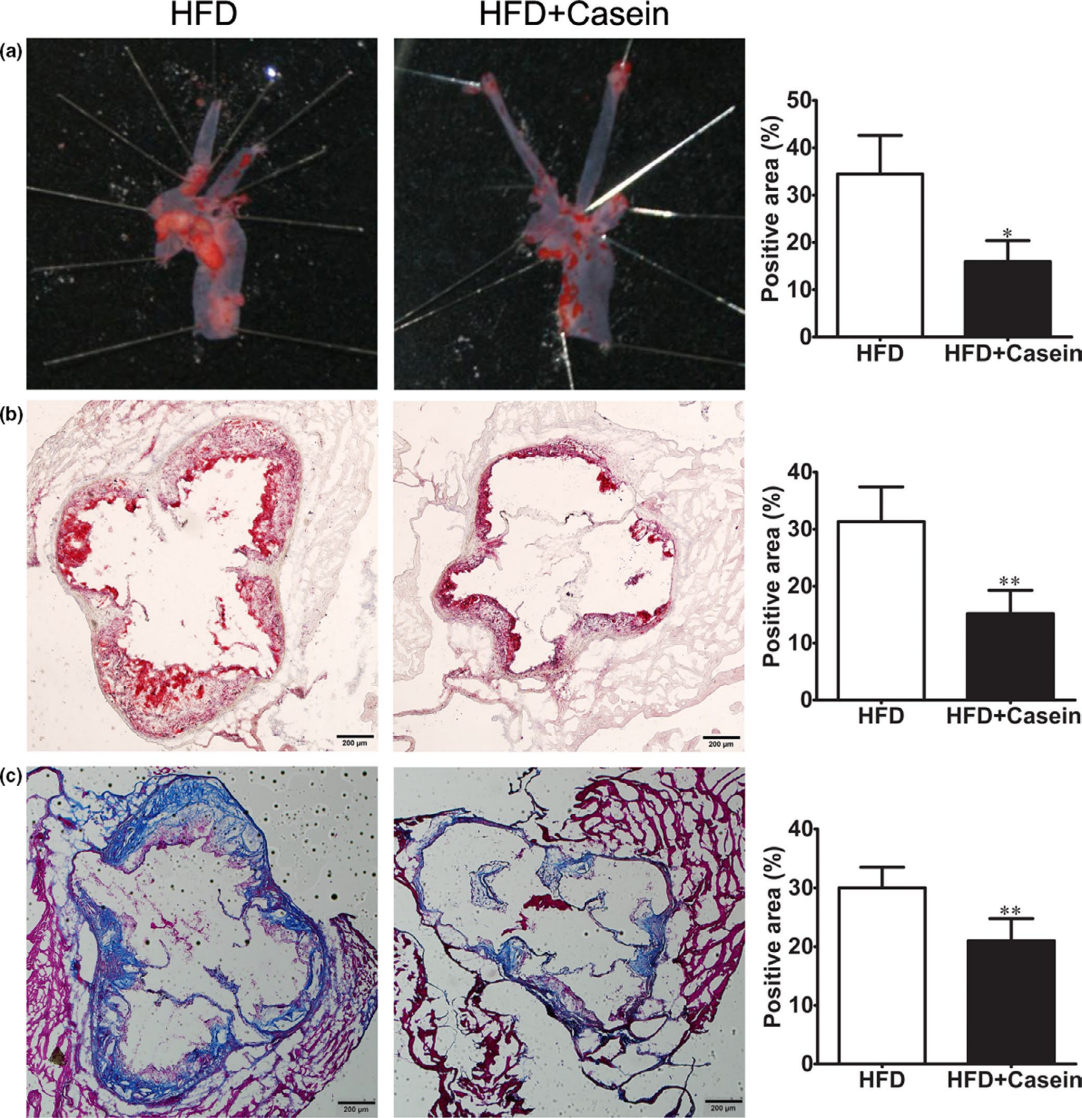
Histological characterization of atherosclerotic plaques in the aortic arch and aortic sinus. ApoE^‐/‐^ mice were fed with high‐fat diet for 12 weeks without (HFD group) or with 10% casein injections (HFD + Casein group). Atherosclerotic plaque areas in the aortic arch and aortic sinus were expressed as positive percentage of the Oil Red O stained area (×40) (A and B, respectively). Collagen deposition in atherosclerotic lesions was expressed as positive percentage of the Masson's Trichrome stained area over the total plaque area (×40) (C). *p < .05, **p < .01

Histological characterization of atherosclerotic plaques in the aortic arch and aortic sinus was shown in Figure 2. Atherosclerotic lesion area was defined as the Oil Red O stained positive area. The positive percentages of atherosclerotic lesion area in aortic arch and aortic sinus were significantly lower in the HFD + Casein group compared with the HFD group (Figure 2a,b). Collagen deposition in atherosclerotic lesions was expressed as the Masson's Trichrome stained positive area. The positive percentage of collagen deposition area in aortic sinus plaques was also significantly reduced in the HFD + Casein group compared with the HFD group (Figure 2c).

### 3.3 Plasma levels of inflammatory markers of the HFD group and HFD + casein group

**FIGURE 3:**
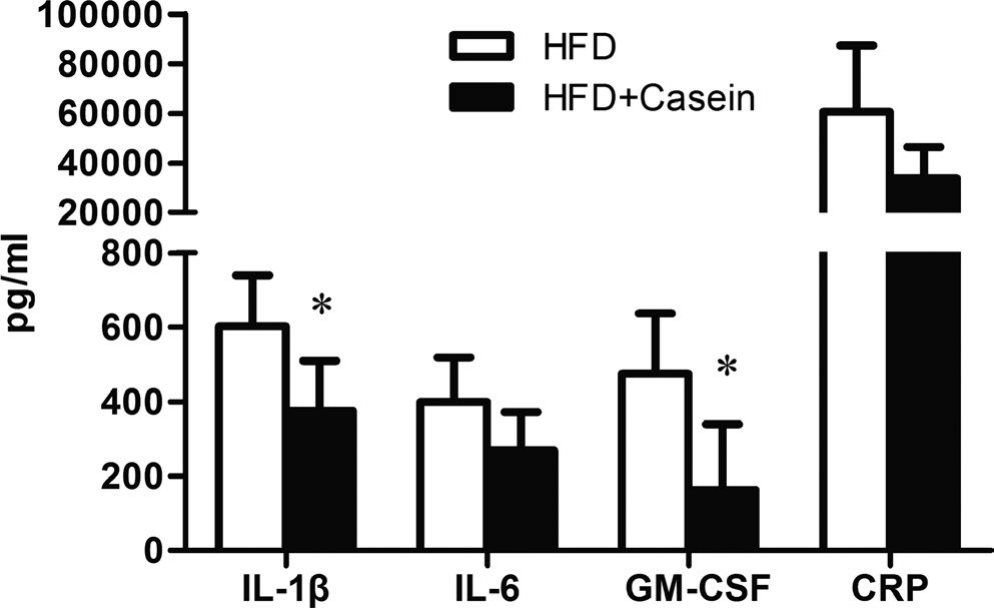
Inflammatory markers analysis in plasma. ApoE^‐/‐^ mice were fed with high‐fat diet for 12 weeks without (HFD group) or with 10% casein injections (HFD + Casein group). Blood samples were collected, and the plasma inflammatory markers were measured. *p < .05

As shown in Figure 3, plasma levels of IL‐1β and GM‐CSF were significantly lower, and IL‐6 and CRP tended to be lower in the HFD + Casein group compared with the HFD group.

## 4 DISCUSSION

The major findings of this study are as follows: (1) 10% casein treatment decreases plasma TC, TG, and LDL‐C concentration and alleviates aortic atherosclerosis in male apoE^‐/‐^ mice fed with HFD. (2) Collagen deposition in aortic sinus plaques and plasma levels of inflammatory markers as IL‐1β and GM‐CSF are reduced after casein intervention. These results indicate that 10% casein treatment slows down atherosclerotic lesion progression, possibly by decreasing fibrosis and inflammatory responses in male apoE^‐/‐^ mice fed with HFD.

### 4.1 The influence of casein on blood lipid level in male apoE^‐/‐^ mice fed with HFD

Atherosclerosis remains the major cause of ischemic vascular diseases. Since the underlying pathological process of ischemic vascular diseases is intravascular fat accumulation leading to atherosclerosis, early intervention in diet and other risk factors might affect the progression of atherosclerosis. It was reported in most studies that casein increased serum cholesterol levels and played a role in the development of atherosclerosis as early as in the 1980s (Huff et al., 1982; Terpstra, Harkes, & van der Veen, 1981). On the contrary, our study demonstrated that casein alleviated hypercholesterolemia in male apoE^‐/‐^ mice fed with HFD, which was in line with previous report by Meinertz et al. (1990). The main reasons might be as follows:

First, an explanation for the discrepancy between our results and those of other authors might be the different species used. The hypercholesterolemia potential of casein would mainly be expressed in species (like the rabbit) with a low intestinal phosphatase activity and with a high glycine conjugation of bile acids. Van der Meer, De Vries, and Van Tintelen (1988) reported that dietary casein increased phosphate absorption and decreased fecal excretion of bile acids in male New Zealand white rabbits, thus increased the free concentration of bile acids and consequently stimulated the reabsorption of steroids from the gut. Eventually, this might cause an increase in serum cholesterol concentration because of the well‐known suppression of hepatic apoB/E receptors (Brown & Goldstein, 1983).

Second, gender appeared to play a role on blood lipid levels and the development of atherosclerosis. Terpstra, Van Tintelen, and West (1982) investigated the effect of dietary casein on the serum concentration of cholesterol in male and female lean Zucker strain rats. The increase of cholesterol level in the serum was observed in the female rats fed the casein diet for 14 weeks during the experiment, whereas the male rats fed the casein diet exhibited a lower serum cholesterol level at the end of the experiment than at the beginning. Similar findings were reported by Fillios, Naito, Andrus, Portman, and Martin (1958) who found that after albino rats of the Sprague–Dawley strain were fed casein diet for a period of 17 weeks, serum cholesterol levels of female rats were twice as high as that of the corresponding males. Atherosclerotic lesions were reported to be larger and more advanced in young female than male apoE^‐/‐^mice because of sex differences in immune mechanisms with activated T cells responding to oxidized LDL in female mice even in the absence of exogenous estrogens, while no such effect was seen in male mice (Caligiuri, Nicoletti, Zhou, Tornberg, & Hansson, 1999). These studies indicated that female animals were more predisposed to developing hypercholesterolemia and atherosclerosis in response to casein diet than their male counterparts.

Third, serum lipid level is quite sensitive to the protein level in the diet. A study in female lean Zucker strain rats showed that the hypercholesterolemic effect of dietary casein could be enhanced by increasing the proportion of this protein in the diet from 20% to 50%. (Terpstra et al., 1982) Furthermore, Huffman and Jones (1956) observed that the level of serum cholesterol in male weanling rats of the Sprague–Dawley stock fed 12.5% casein for 25 weeks was significantly lower than in those fed 25%, 9%, or 7.5% and tended to be lower than in those fed 18.75%, which revealed that reduction or elevation of dietary casein beyond a modest concentration (12%–18%) might lead to ultimate elevation of serum cholesterol. This observation was supported by Fillios et al. (1958) who reported that inadequate (5%) or excessive (40% or 60%) levels of dietary casein were found to contribute to an increased degree of serum cholesterol in male albino rats of the Sprague–Dawley strain. Regulation of plasma TG level is primarily mediated by lipoprotein lipase (LPL) attached to the capillary endothelium which catalyzes the hydrolytic cleavage of TG into fatty acids (Kersten, 2014). A study in male Wistar rats confirmed that tissue LPL activities were decreased with the consumption of a low‐protein diet containing 50 g/kg protein compared with a balanced diet containing 200 g/kg protein (Boualga, Bouchenak, & Belleville, 2000).As a consequence, inadequate levels of dietary protein followed by decreased LPL activity could increase the levels of TG in plasma. These studies explained that too high or too low proportion of casein in dietary could increase the levels of serum lipids.

Taken together, the results from above studies might partially explain inconsistent effects of casein on serum cholesterol levels. Therefore, it was reasonable for 10% casein to reduce blood lipid levels in male apoE^‐/‐^ mice fed with HFD in this study. Our study provides a potential way to alleviate hyperlipidemia induced by HFD in some individuals through administration of appropriate casein proportion.

### 4.2 Potential mechanisms of casein affecting atherosclerotic progression

Vascular fibrosis, involving accumulation of extracellular matrix proteins particularly collagen, is associated with atherosclerosis. Previous study showed that vascular fibrosis contributed to vascular remodeling and scar formation (Touyz, 2005). Arterial stiffening or remodeling was recognized as an important consequence of vascular fibrosis to promote the development of atherosclerosis (Shirwany & Zou, 2010). The results of present study demonstrated that atherosclerotic plaque area and collagen deposition in the aortic root were significantly decreased after casein treatment, suggesting that casein reduced the progression of vascular fibrosis and thereby attenuated atherosclerotic development in male apoE^‐/‐^ mice fed with HFD.

The induction of inflammation provides the important link between hyperlipidemia and atherosclerosis. During the past decades, extensive observations supported a central role for inflammation in all phases of atherosclerotic process, including early atherogenesis, lesion progression, and final thrombotic complications (Pant et al., 2014). In a variety of animal models of atherosclerosis, signs of inflammation occur hand in hand with incipient lipid accumulation in the artery wall. Once adherent to the endothelium, the blood‐derived leukocytes migrate into the intima at sites of lesion formation, participate in and perpetuate a local inflammatory response. The activated macrophage differentiated from entering monocytes could produce proteolytic enzymes rendering the plaque's protective fibrous cap thin, weak, and susceptible to rupture, which triggers the thrombus that causes most acute complications of atherosclerosis. Accumulating evidence indicated that elevated circulating inflammatory markers reflected the local inflammatory process for atherosclerosis and predicted adverse cardiovascular events (Pant et al., 2014). Our study showed that the reduction of plaque area in the aortic arch and aortic sinus was accompanied with lowering of plasma inflammatory markers in male apoE^‐/‐^ mice following casein injection, which indicated the beneficial cardiovascular protective effect of casein on atherosclerotic development by decreasing inflammatory responses.

### 4.3 Study limitations

The influences of casein on atherosclerotic development are varied in different gender and doses. This study only demonstrated that casein (subcutaneous injection with 0.5 ml of 10% casein daily for 12 weeks) attenuated atherosclerotic development in male apoE^‐/‐^ mice fed with HFD (1.25% cholesterol). Future studies are warranted to explore dose dependent effect of casein on atherosclerotic progression and its potential mechanism in both male and female apoE^‐/‐^ mice. It is to note that present study gave casein to mouse by subcutaneous injection, which is different from previous studies and is also different from casein's daily oral consumption habits; therefore, the results of subcutaneous injection cannot be compared with the results of previous oral consumption.

In conclusion, our study finds that 10% casein treatment decreases lipid concentrations in male apoE^‐/‐^ mice fed with HFD. Casein administration is associated with attenuated atherosclerotic lesion progression, possibly by decreasing fibrosis and inflammatory responses in male apoE^‐/‐^ mice fed with HFD.

## 5 ETHICAL REVIEW

This study was approved by the Animal Care Committees of Wuhan Fourth Hospital.

## CONFLICT OF INTEREST

The authors declare that they do not have any conflict of interest.

## References

[fsn31638-bib-0001] Bohl, M. , Bjornshave, A. , Gregersen, S. , & Hermansen, K. (2016). Whey and casein proteins and medium‐chain saturated fatty acids from milk do not increase low‐grade inflammation in abdominally obese adults. The Review of Diabetic Studies, 13(2–3), 148–157. 28012280 10.1900/RDS.2016.13.148 PMC5553764

[fsn31638-bib-0002] Boualga, A. , Bouchenak, M. , & Belleville, J. (2000). Low‐protein diet prevents tissue lipoprotein lipase activity increase in growing rats. British Journal of Nutrition, 84(5), 663–671. 11177179 10.1017/s0007114500002002

[fsn31638-bib-0003] Brown, M. S. , & Goldstein, J. L. (1983). Lipoprotein receptors in the liver. Control signals for plasma cholesterol traffic. Journal of Clinical Investigation, 72(3), 743–747. 6309907 10.1172/JCI111044 PMC1129238

[fsn31638-bib-0004] Burhans, M. S. , Hagman, D. K. , Kuzma, J. N. , Schmidt, K. A. , & Kratz, M. (2018). Contribution of adipose tissue inflammation to the development of Type 2 diabetes mellitus. Comprehensive Physiology, 9(1), 1–58. 30549014 10.1002/cphy.c170040 PMC6557583

[fsn31638-bib-0005] Cadee, J. A. , Chang, C. Y. , Chen, C. W. , Huang, C. N. , Chen, S. L. , & Wang, C. K. (2007). Bovine casein hydrolysate (c12 Peptide) reduces blood pressure in prehypertensive subjects. American Journal of Hypertension, 20(1), 1–5. 17198904 10.1016/j.amjhyper.2006.06.005

[fsn31638-bib-0006] Caligiuri, G. , Nicoletti, A. , Zhou, X. , Tornberg, I. , & Hansson, G. K. (1999). Effects of sex and age on atherosclerosis and autoimmunity in apoE‐deficient mice. Atherosclerosis, 145(2), 301–308. 10488957 10.1016/s0021-9150(99)00081-7

[fsn31638-bib-0007] Cox, A. J. , West, N. P. , & Cripps, A. W. (2015). Obesity, inflammation, and the gut microbiota. Lancet Diabetes & Endocrinology, 3(3), 207–215. 25066177 10.1016/S2213-8587(14)70134-2

[fsn31638-bib-0008] Cross, M. L. , & Gill, H. S. (2000). Immunomodulatory properties of milk. British Journal of Nutrition, 84(Suppl 1), S81–89. 11242451 10.1017/s0007114500002294

[fsn31638-bib-0009] Dalziel, J. E. , Young, W. , McKenzie, C. M. , Haggarty, N. W. , & Roy, N. C. (2017). Gastric emptying and gastrointestinal transit compared among native and hydrolyzed whey and casein milk proteins in an aged rat model. Nutrients, 9(12), 1351. 10.3390/nu9121351 PMC5748801 29236034

[fsn31638-bib-0010] Damasceno, N. R. , Gidlund, M. A. , Goto, H. , Dias, C. T. , Okawabata, F. S. , & Abdalla, D. S. (2001). Casein and soy protein isolate in experimental atherosclerosis: Influence on hyperlipidemia and lipoprotein oxidation. Annals of Nutrition & Metabolism, 45(1), 38–46. 11244186 10.1159/000046704

[fsn31638-bib-0011] Elwood, P. C. , Pickering, J. E. , Givens, D. I. , & Gallacher, J. E. (2010). The consumption of milk and dairy foods and the incidence of vascular disease and diabetes: An overview of the evidence. Lipids, 45(10), 925–939. 10.1007/s11745-010-3412-5 20397059 PMC2950929

[fsn31638-bib-0012] Fillios, L. C. , Naito, C. , Andrus, S. B. , Portman, O. W. , & Martin, R. S. (1958). Variations in cardiovascular sudanophilia with changes in the dietary level of protein. American Journal of Physiology, 194(2), 275–279. 10.1152/ajplegacy.1958.194.2.275 13559462

[fsn31638-bib-0013] Gao, B. , Li, L. , Zhu, P. , Zhang, M. , Hou, L. , Sun, Y. , … Gu, Y. (2015). Chronic administration of methamphetamine promotes atherosclerosis formation in ApoE‐/‐ knockout mice fed normal diet. Atherosclerosis, 243(1), 268–277. 10.1016/j.atherosclerosis.2015.09.001 26409626

[fsn31638-bib-0014] Hedin, U. , & Matic, L. P. (2018). Recent advances in therapeutic targeting of inflammation in atherosclerosis. Journal of Vascular Surgery, 69(3), 944–951. 30591299 10.1016/j.jvs.2018.10.051

[fsn31638-bib-0015] Huff, M. W. , Roberts, D. C. , & Carroll, K. K. (1982). Long‐term effects of semipurified diets containing casein or soy protein isolate on atherosclerosis and plasma lipoproteins in rabbits. Atherosclerosis, 41(2–3), 327–336. 10.1016/0021-9150(82)90197-6 7199927

[fsn31638-bib-0016] Huffman, S. , & Jones, R. J. (1956). Chronic effect of dietary protein on hypercholesteremia in the rat. Proceedings of the Society for Experimental Biology and Medicine, 93(3), 519–522. 10.3181/00379727-93-22804 13389507

[fsn31638-bib-0017] Ijaz, M. U. , Ahmed, M. I. , Zou, X. , Hussain, M. , Zhang, M. , Zhao, F. , … Li, C. (2018). Beef, casein, and soy proteins differentially affect lipid metabolism, triglycerides accumulation and gut microbiota of high‐fat diet‐fed C57BL/6J mice. Frontiers in Microbiology, 9, 2200 10.3389/fmicb.2018.02200 30319558 PMC6165900

[fsn31638-bib-0018] Kersten, S. (2014). Physiological regulation of lipoprotein lipase. Biochimica Et Biophysica Acta, 1841(7), 919–933. 10.1016/j.bbalip.2014.03.013 24721265

[fsn31638-bib-0019] Lillefosse, H. H. , Tastesen, H. S. , Du, Z. Y. , Ditlev, D. B. , Thorsen, F. A. , Madsen, L. , … Liaset, B. (2013). Hydrolyzed casein reduces diet‐induced obesity in male C57BL/6J mice. Journal of Nutrition, 143(9), 1367–1375. 10.3945/jn.112.170415 23843475

[fsn31638-bib-0020] Lira, F. S. , Rosa Neto, J. C. , Antunes, B. M. , & Fernandes, R. A. (2014). The relationship between inflammation, dyslipidemia and physical exercise: From the epidemiological to molecular approach. Current Diabetes Review, 10(6), 391–396. 10.2174/1573399810666141122210135 25418583

[fsn31638-bib-0021] Lontchi‐Yimagou, E. , Sobngwi, E. , Matsha, T. E. , & Kengne, A. P. (2013). Diabetes mellitus and inflammation. Current Diabetes Reports, 13(3), 435–444. 10.1007/s11892-013-0375-y 23494755

[fsn31638-bib-0022] McGregor, R. A. , & Poppitt, S. D. (2013). Milk protein for improved metabolic health: A review of the evidence. Nutrition & Metabolism, 10(1), 46 10.1186/1743-7075-10-46 23822206 PMC3703276

[fsn31638-bib-0023] Meinertz, H. , Nilausen, K. , & Faergeman, O. (1990). Effects of dietary proteins on plasma lipoprotein levels in normal subjects: Interaction with dietary cholesterol. Journal of Nutritional Science and Vitaminology, 36(Suppl 2), S157–164. 10.3177/jnsv.36.SupplementII_S157 2130150

[fsn31638-bib-0024] Pal, S. , & Ellis, V. (2010). The chronic effects of whey proteins on blood pressure, vascular function, and inflammatory markers in overweight individuals. Obesity (Silver Spring), 18(7), 1354–1359. 10.1038/oby.2009.397 19893505

[fsn31638-bib-0025] Panagiotakos, D. B. , Pitsavos, C. H. , Zampelas, A. D. , Chrysohoou, C. A. , & Stefanadis, C. I. (2010). Dairy products consumption is associated with decreased levels of inflammatory markers related to cardiovascular disease in apparently healthy adults: The ATTICA study. Journal of the American College of Nutrition, 29(4), 357–364. 10.1080/07315724.2010.10719852 21041810

[fsn31638-bib-0026] Pant, S. , Deshmukh, A. , Gurumurthy, G. S. , Pothineni, N. V. , Watts, T. E. , Romeo, F. , & Mehta, J. L. (2014). Inflammation and atherosclerosis–revisited. Journal of Cardiovascular Pharmacology and Therapeutics, 19(2), 170–178. 10.1177/1074248413504994 24177335

[fsn31638-bib-0027] Pasin, G. , & Comerford, K. B. (2015). Dairy foods and dairy proteins in the management of type 2 diabetes: A systematic review of the clinical evidence. Advances in Nutrition, 6(3), 245–259. 10.3945/an.114.007690 25979490 PMC4424779

[fsn31638-bib-0028] Saito, T. (2008). Antihypertensive peptides derived from bovine casein and whey proteins. Advances in Experimental Medicine and Biology, 606, 295–317. 18183935 10.1007/978-0-387-74087-4_12

[fsn31638-bib-0029] Sanchez, D. , Kassan, M. , Contreras Mdel, M. , Carron, R. , Recio, I. , Montero, M. J. , & Sevilla, M. A. (2011). Long‐term intake of a milk casein hydrolysate attenuates the development of hypertension and involves cardiovascular benefits. Pharmacological Research, 63(5), 398–404. 10.1016/j.phrs.2011.01.015 21300153

[fsn31638-bib-0030] Shirwany, N. A. , & Zou, M. H. (2010). Arterial stiffness: A brief review. Acta Pharmacologica Sinica, 31(10), 1267–1276. 10.1038/aps.2010.123 20802505 PMC3078647

[fsn31638-bib-0031] Terpstra, A. H. , Harkes, L. , & van der Veen, F. H. (1981). The effect of different proportions of casein in semipurified diets on the concentration of serum cholesterol and the lipoprotein composition in rabbits. Lipids, 16(2), 114–119. 10.1007/BF02535684 6941061

[fsn31638-bib-0032] Terpstra, A. H. , Van Tintelen, G. , & West, C. E. (1982). The effect of semipurified diets containing different proportions of either casein or soybean protein on the concentration of cholesterol in whole serum, serum lipoproteins and liver in male and female rats. Atherosclerosis, 42(1), 85–95. 10.1016/0021-9150(82)90129-0 7200791

[fsn31638-bib-0033] Torres, N. , Guevara‐Cruz, M. , Velazquez‐Villegas, L. A. , & Tovar, A. R. (2015). Nutrition and Atherosclerosis. Archives of Medical Research, 46(5), 408–426. 10.1016/j.arcmed.2015.05.010 26031780

[fsn31638-bib-0034] Touyz, R. M. (2005). Intracellular mechanisms involved in vascular remodelling of resistance arteries in hypertension: Role of angiotensin II. Experimental Physiology, 90(4), 449–455. 10.1113/expphysiol.2005.030080 15890798

[fsn31638-bib-0035] Van der Meer, R. , De Vries, H. T. , & Van Tintelen, G. (1988). The phosphorylation state of casein and the species‐dependency of its hypercholesterolaemic effect. British Journal of Nutrition, 59(3), 467–473. 10.1079/BJN19880056 3395606

[fsn31638-bib-0036] Xu, J. Y. , Qin, L. Q. , Wang, P. Y. , Li, W. , & Chang, C. (2008). Effect of milk tripeptides on blood pressure: A meta‐analysis of randomized controlled trials. Nutrition, 24(10), 933–940. 10.1016/j.nut.2008.04.004 18562172

